# High Adenoma Detection Rates in Fecal Immunochemical Test-Based Colorectal Cancer Screening: Interim Results of the National Bowel Cancer Screening Program in Qatar

**DOI:** 10.7759/cureus.32274

**Published:** 2022-12-06

**Authors:** Anil K John, Betsy Varughese, Shaikha S Abushaikha, Ahed M Hamdan, Viswapriya Pillai, Ahmad M Ayash, Paul K Vincent, Khaleel Sultan, Khalid M Al Ejji, Rajvir Singh, Samya Alabdulla, Mariam Abdulmalik, Saad Al Kaabi

**Affiliations:** 1 Gastroenterology and Hepatology, Hamad Medical Corporation, Doha, QAT; 2 Community Medicine, Primary Health Care Corporation, Doha, QAT; 3 Preventative Health Screening Programs, Primary Health Care Corporation, Doha, QAT; 4 Cardiology Research, Heart Hospital, Hamad Medical Corporation, Doha, QAT; 5 Family Medicine, Primary Health Care Corporation, Doha, QAT

**Keywords:** crc, adenoma detection, bowel cancer screening, colorectal cancer, fecal immunochemical test

## Abstract

Introduction: Colorectal cancer is one of the most common cancers globally. Recent reductions in mortality rates have been primarily attributed to screening programs. The State of Qatar established a national bowel cancer screening program in 2016.

Methodology: Fecal immunochemical testing (FIT) was used for average-risk individuals aged 50 to 74 years. Fecal immunochemical testing -positive participants were referred for total colonoscopy to detect polyps and cancers.

Results: Among 32,751 FIT invitees, 11,130 took the test, and 758 (6%) of those were FIT positive. Of these, 375 (56.13%) participants underwent a colonoscopy, and polyps were detected in 198 (52.8%) and cancers in 19 (5.1%) participants. The adenoma detection rate exceeded 40%.

Discussion and conclusion: The high yield of polyps and cancers in the screening program justifies an active, resource-intensive, and organized bowel cancer screening effort. The high adenoma detection rate in a FIT-based program warrants recalibration of target adenoma detection rates in screening programs.

## Introduction

Colorectal cancer (CRC) is the third most common cancer in the world [1]. Although CRC incidence and mortality rates have decreased in the United States and most European countries [2], a paradoxical increase in CRC incidence and mortality has been observed in low-income countries [3]. The five-year survival of CRC in developing countries is markedly lower than in the developed world, where survival is well over 60% [4]. The primary reason for this reduced CRC mortality can be attributed to screening programs [5]. There is a substantial research evidence that shows the benefits of early detection of cancer and removal of polyps in asymptomatic individuals [6]. However, widespread implementation of an organized screening program has been undertaken in only a few countries, mostly in Europe and North America [7,8], due to the cost and resources required [9].

Based on the results of a pilot study demonstrating a significant number of cancers and adenomatous polyps in average-risk asymptomatic individuals aged 50 to 74 [10], in 2016, the State of Qatar implemented a national population-based CRC screening program under the Supreme Council of Health and organized by the Primary Health Center Corporation (PHCC). Program guidelines recommend a fecal immunochemical test (FIT) every two years for asymptomatic, average-risk individuals in the target age group. In this study, we audited colonoscopy outcomes among average-risk individuals who participated in this screening and were referred to our colonoscopy unit with a positive FIT.

## Materials and methods

Participants included those who received a positive FIT under the national screening program undertaken by the Supreme Council of Health in the State of Qatar. All were average-risk asymptomatic individuals aged 50 to 74 years and were referred to the endoscopy unit for total optical colonoscopy. Those with gastrointestinal symptoms or personal or family history of colonic cancer or polyps were excluded and referred for direct colonoscopy outside the average risk pathway. Those with significant debilitating comorbidities with an expected life expectancy of <10 years also were excluded.

For the FIT procedure, participants were asked to send three fecal samples from consecutive bowel movements according to the manufacturer’s instructions of a commercially available FIT kit (OC-Auto 3 Latex Reagent, Eiken Chemicals Co. Ltd., Japan). A test was considered positive if at least one of the samples showed >20 µg/g (>100 ng/mL) hemoglobin per gram of feces. Participants with positive tests were referred for total colonoscopy within 30 days at the Ambulatory Care Center of Hamad Medical Corporation in Doha, Qatar. Those with a negative FIT test would be asked to repeat the FIT test in two years as per the biennial screening protocol at the PHCC level. Those with a negative FIT test would be asked to repeat the FIT test in two years as per the biennial screening protocol at the PHCC level. Those with a negative colonoscopy and nil family history are waived from further screening for the next 10 years. 

Colonoscopies were conducted by a staff endoscopist consultant or specialist or by a trainee endoscopist supervised by a consultant. Bowel preparation for the screening colonoscopy was deemed good if no residual fecal matter was seen and fair if fecal residue was washable. If fecal matter precluded optimal examination, the preparation was considered poor and the procedure was abandoned. A histopathological examination of any specimens was conducted by a specialist gastrointestinal pathologist.

The primary outcome was the detection of cancers or polyps. Secondary outcomes included monitoring adherence to the key performance indicators of the program and auditing any adverse events during or after the colonoscopy.

All patients undergoing colonoscopy received an American Society of Anesthesiologists (ASA) score [11] from the physician before the procedure. Those in the ASA class I to II category underwent colonoscopy under conscious sedation using titrated doses of midazolam and fentanyl, administered by the endoscopist. Those with ASA scores of III or higher had assistance from an anesthetist for the procedure whenever required.

Any polyps detected during colonoscopy were removed when possible, and any suspicious lesions were biopsied. In patients with multiple polyps, the most significant polyp in terms of size, adenomatous, or villous features was considered for analysis. The overall adenoma detection rate (ADR) also was calculated. The ADR is defined as the detection of at least one adenomatous polyp in a screening colonoscopy and is considered a robust quality matrix of screening colonoscopy [12].

## Results

Of the 32,751 FIT invitations sent by participating primary health centers, 11,130 returned the FIT kit results. Of these, 758 (6.8%) were FIT-positive. After excluding those with significant comorbidities and other exclusions, 668 average-risk participants aged 50 to 74 (median: 57.60 years) were included in the study. Finally, 375 (56.13%; 56.3% female) underwent colonoscopies from January 2018 to January 2019, 251 (66.9%) of which were performed within 30 days of the referral. Table 1 summarizes the demographic and colonoscopy outcomes.

**Table 1:** Demography and colonoscopy outcomes FIT: Fecal immunochemical test, CI: Confidence interval, PPV: Positive predictive value

Variable	N (%)
Number of invitations sent for FIT	32457
Number of individuals tested for FIT	11130
Number of FIT-positive individuals	758 (6.8%)
Number of FIT-positive individuals referred to colonoscopy unit	668
Total colonoscopy uptake	375 (56.13%)
Age of the participants (Years)	57.60 ± 6.07
Gender (Male)	164 (43.7%)
Cecal Intubation Rate (CIR)	364 (97.1 %)
Optimal bowel preparation	368 (98.1%)
Colonoscopy within 30 days of positive FIT	251 (66.9%)
Overall adenoma detection rate	40.8%
Positive predictive value for colorectal cancers (PPV, 95% CI)	5.1%, (3.1% -7.8%)
Positive predictive value for adenoma (PPV, 95% CI)	40.8%, (36.0% - 46.0%)

Cecal intubation for total colonoscopy was performed in 364 (97.1%) participants. Bowel preparation was good in 368 (98.1%) and fair in seven (1.9%) individuals, enabling optimal examination for 375 patients. A minimum of seven minutes of withdrawal time was ensured in all colonoscopies. Of all patients who underwent colonoscopy, 19 (5.1%) had cancer proven by histopathology, and 198 (52.8%) had polyps. Of these polyps, 150 were adenomatous and three were serrated adenomas, indicating neoplastic nature; 38 polyps were hyperplastic, and seven were inflammatory. For the remaining participants, 63 (16.8%) had a normal colonoscopy and 95 (25.3%) had incidental findings (e.g., hemorrhoids, colonic ulcers, inflammatory bowel disease, and diverticulosis). The calculated ADR was 40.8% (53.7% in males and 30.8% in females) in our study, which exceeds the accepted international benchmark. Most cancers detected were amenable to treatment, and their American Joint Committee on Cancer stages varied from I to III [13].

## Discussion

Colorectal cancer is one of the few cancers that fulfills most screening criteria as per the gold standard established by Wilson and Jungner [14]. It has a high incidence, a long preclinical phase, and treatable precursor lesions making screening even more important. Colorectal cancer screening is cost-effective compared to no screening [15]. Screening programs define the average risk for CRC for individuals aged 50 years or older with no additional risk factors. Most guidelines recommend annual or biennial FIT, sigmoidoscopy every five years, or colonoscopy every 10 years [16].

In Qatar, the modality for organized, programmatic, population-based screening is the fecal immunochemical test at the PHCC level. Systematic reviews have established reductions in the relative risk of CRC mortality using fecal occult blood tests [17]. The follow-up data on these participants shows that the positive effects of screening persist in the long term [18]. The reduction in relative risk of CRC mortality is attributed mostly to the removal of precursor adenomatous polyps and early detection of cancer, and the benefit is more marked in those who receive annual screening, compared to biennial screening [19].

Fecal occult blood tests include FIT-based and guiac-based (g-FOBT) testing. Compared to guiac-based testing, FIT performs better at detecting advanced adenomas and CRC [20]. Screening programs using FIT also have higher participation rates [21], likely because FIT does not require any dietary restrictions. The FIT gives quantitative results (microgram hemoglobin per gram feces) and automated reading of results. The optimal cut-off value of FIT should be based on the availability of resources, epidemiology of CRC in the population, and participation rates [22]. In our study, a positive FIT was any result showing >20 µg/g (>100 ng/mL) of hemoglobin per gram of feces. Values between 20 to 50 µg of hemoglobin per gram of feces are recommended for screening when the healthcare system can accommodate a 5% rate of FIT positivity in the total population screened [23]. Today, FIT is the first option for fecal occult blood testing in organized CRC screening programs [24]. In Qatar, the PHCC adopted FIT as the primary screening tool based on current evidence. 

The National Bowel Cancer Screening Program of the Supreme Council of Health in Qatar is an organized screening program with an explicit policy, specified age category, methods, target population, management team, and quality assurance and monitoring structures as per the International Agency for Research in Cancer [25]. In contrast, an opportunistic screening program is delivered by a physician on a case-by-case basis to eligible participants. Organized screening programs typically have stronger quality assurance structures and higher participation rates than opportunistic ones [26].

Quality assurance in colonoscopy is pivotal to any screening program because it is a highly operator-dependent procedure. Most post-colonoscopy colorectal cancers develop from neoplastic lesions that harbor genetic features associated with a more rapid progression to cancer, but many of these cancers are attributed to missed polyps or incompletely resected polyps without adequate surveillance. Evidence has shown a strong association between the quality of the colonoscopy and the rate of interval cancers [27]. The ADR, which is the proportion of patients undergoing screening colonoscopy in whom at least one adenoma is detected, is a robust measure of colonoscopy performance quality [28]. Revision to US Multi-Society Task Force recommendations on ADR measurements in 2015 specified the minimum threshold for adenoma detection rate as 25% overall with 30% in males and 20% in females for any screening colonoscopy [29]. The ADR also has been validated as a robust measure of CRC screening quality [30]. A 1% increase in ADR can contribute to a 3% reduction in the incidence of CRC and a 5% reduction in mortality of CRC [31]. So, ADR monitoring is an essential requirement of any organized screening program.

Over the past decade, FIT-based CRC screening has been implemented worldwide [32]. It is now the most commonly used primary screening method [33]. Participants with a positive FIT have a high prevalence of adenomas, leading to high ADRs for colonoscopies, which explains the ADR exceeding 40% in our study. The FIT-based screening programs also identify participants with an enriched adenoma prevalence for colonoscopy [34]. The median ADR for a primary screening colonoscopy is around 25% compared to around 50% for a screening colonoscopy in FIT-positive populations [30]. Hence, our study adds further evidence to the observation of higher ADR in FIT-positive screening colonoscopy.

The cecal intubation rate is the passage of the colonoscope tip into the cecal caput, permitting full evaluation of the mucosa between the ileocecal valve and the appendiceal orifice. Cecal intubation should be documented via images of the appendiceal orifice, ileocecal valve, and terminal ileum if intubated [35]. The cecal intubation rate is an indicator of a complete examination of the colon, which is a fundamental objective of colonoscopy and is considered a key performance indicator in our program. The current benchmark recommendation for the cecal intubation rate is to exceed 95% of the screening population [36], which we achieved in our study.

Only around 5% of cancers detected in this study were stage IV, implying that most screen-detected cancers were in early stages and amenable to treatment. It is well-documented that screen-detected cancers are predominantly early-stage cancers with better prognoses [37].

For our analysis, we defined adverse events as any event that prevents completion of a planned colonoscopy procedure (excluding poor preparation or technical failure), results in hospital admission, or requires another interventional (e.g., surgical or radiological) procedure beyond endoscopy within 30 days of the index colonoscopy. None of our screening participants met these criteria for adverse events.

An important limitation in our audit was that only around one-third of the FIT invitees in the target population responded to the invitation for screening. Also, colonoscopy uptake among the FIT-positive participants was less than 60%, which limited the overall coverage and inclusiveness of our screening program. We plan to rectify this shortfall in subsequent screening rounds. Our audit of FIT-positive colonoscopy shows a high yield of adenomas, with an ADR exceeding 40% and a cancer rate of 5%. Colonoscopy in a FIT-positive participant thereby implies a 50% probability of polyps and a 5% probability of cancer. The possibility of a normal colonoscopy in a FIT-positive average-risk participant in our study is only around 20%, compared to a 25% probability of other significant findings apart from cancers or polyps.

## Conclusions

In this study, an organized CRC screening program using FIT in average-risk asymptomatic participants yielded a significant number of polyps and cancers with a high ADR that exceeded conventional benchmarks. Our results add to the current evidence of higher ADR in FIT-positive screening colonoscopies. The feasibility of an organized CRC screening program for targeted populations with adequate quality assurance and management structure should be explored in developing nations where such programs are scarce.
